# The effect of shifting medical coverage from National Health Insurance to Medical Aid type I and type II on health care utilization and out-of-pocket spending in South Korea

**DOI:** 10.1186/s12913-020-05778-2

**Published:** 2020-10-27

**Authors:** Doo Woong Lee, Jieun Jang, Dong-Woo Choi, Sung-In Jang, Eun-Cheol Park

**Affiliations:** 1 Department of Public Health, Graduate School, Yonsei University, Seoul, 03722 Republic of Korea; 2 Department of Preventive Medicine, Ajou University School of Medicine, Suwon, 16499 Republic of Korea; 3 Institute of Health Services Research, Yonsei University, Seoul, 03722 Republic of Korea; 4 Department of Preventive Medicine, Yonsei University College of Medicine, Seoul, 03722 Republic of Korea

**Keywords:** South Korea, Medical aid, National Health Insurance, Health care utilization, Out-of-pocket medical spending, Difference-in-differences, Propensity score

## Abstract

**Background:**

This study examines the effects of a shift in medical coverage, from National Health Insurance (NHI) to Medical Aid (MA), on health care utilization (measured by the number of outpatient visits and length of stay; LOS) and out-of-pocket medical expenses.

**Methods:**

Data were collected from the Korean Welfare Panel Study (2010–2016). A total of 888 MA Type I beneficiaries and 221 MA Type II beneficiaries who shifted from the NHI were included as the case group and 2664 and 663 consecutive NHI holders (1:3 propensity score-matched) were included as the control group, respectively. We used the ‘difference-in-differences’ (DiD) analysis approach to assess changes in health care utilization and medical spending by the group members.

**Results:**

Differential average changes in outpatient visits in the MA Type I panel between the pre- and post-shift periods were significant, but differential changes in LOS were not found. Those who shifted from NHI to MA Type I had increased number of outpatient visits without changes in out-of-pocket spending, compared to consecutive NHI holder who had similar characteristics. However, this was not found for MA Type II beneficiaries.

**Conclusion:**

Our research provides evidence that the shift in medical coverage from NHI to MA Type I increased the number of outpatient visits without increasing the out-of-pocket spending. Considering the problem of excess medical utilization by Korean MA Type I beneficiaries, further researches are required to have in-depth discussions on the appropriateness of the current cost-sharing level on MA beneficiaries.

## Background

South Korea has often been acclaimed for providing universal medical coverage for its entire population in only 12 years [1]. The Korean National Health Insurance (NHI) system began by providing cover for industrial workers in large corporations in 1977. It was gradually extended to other groups like self-employed workers until the scheme covered the entire population by 1989. Along with the NHI, a Medical Aid (MA) program was simultaneously initiated in 1977 as part of a South Korean social welfare program, called the National Basic Livelihood Security System, which supports poor people in need of medical assistance. It is comparable to the USA’s Medicaid program.

Approximately 3 to 4% of the entire population are entitled to MA, and they are segregated into Type I and Type II recipients based on their economic inability or incapacitation (2.8% in 2017) [2]. Type I covers those who are socially deprived and or incapable of working (those aged under 18 or over 65; disabled people; those with severe and rare diseases, and other special cases) [3, 4]. Type II also covers those who are socially deprived but are capable of working [3, 4]. Type I beneficiaries are not required to provide copayments for any medical utilization, whereas Type II beneficiaries have minimum copayment rates of up to 15% [3, 4].

Over time, the MA program has undergone many modifications regarding its beneficiary inclusion criteria and coverage expansion plan, along with the challenges of its sustainability. In 2006, the Korean government announced the need for a major amendment to the MA law owing to the challenges faced in continuing the MA program. These challenges arose from the increase in the number of beneficiaries and the expansion of services; the increased incidence of chronic diseases and an aging population resulted in the continuous rise in reimbursements for MA beneficiaries [3]. Furthermore, about 10% of the beneficiaries used health care services excessively and accounted for about 60% of the total MA expenditure [3, 5]. Additionally, the moral hazards of MA utilization by MA beneficiaries were publicized continuously, because as health care spending increased with insurance, the real value of health care declined compared to the costs incurred in providing it [5].

Accordingly, in 2007, the Korean government implemented several cost-sharing directives. First, the government mandated out-of-pocket spending on outpatient services for Type I beneficiaries [6]. Besides, since 2018, Type I beneficiaries have had to pay ₩1000, ₩2000, and ₩3000 ($1 = around ₩1120) for each outpatient visit to a clinic, secondary hospital, and tertiary hospital, respectively, while there is no cost for inpatient services availed [7]. Furthermore, Type II beneficiaries must pay ₩1000 per outpatient clinic visit and 15% extra for a single secondary or tertiary hospital outpatient visit; and there is an out-of-pocket expense of 10% of the total expenditure for inpatient services availed [7].

In addition to mandating out-of-pocket spending by beneficiaries, the government levied a monthly health management fee to moderate possible abuse of medical facilities provided under the Healthy Life Maintenance Aid Program [7, 8]. Through this program, each Type I beneficiary receives, ₩6000 (around $6) monthly, via a virtual account [7, 8]. Upon receipt of outpatient medical services, beneficiaries make a copayment via the virtual account [7, 8]. If the beneficiaries spend the entire amount available in the virtual account, they must bear the additional costs themselves [7, 8]. Money remaining in the virtual account cannot be converted to cash [7, 8].

Despite these changes, the total medical expenditure by MA beneficiaries has steadily increased, and the medical costs per person are three times higher than for NHI covered individuals [8–13]. Furthermore, several recent studies have revealed that MA beneficiaries use outpatient services more frequently and stay longer in hospital compared with NHI-covered individuals [8, 10–13]. Studies have also revealed the differences between the health care utilization of MA and NHI beneficiaries. It has been observed that MA Type I and Type II beneficiaries share some socioeconomic status (SES) characteristics, such as age, income, health status, and economic activity status [9]. Few studies have compared health care utilization between MA beneficiaries and NHI-covered individuals with similar SES [12, 13]. However, these studies have neither compared groups with changing health care utilization, nor medical spending, and they have not considered the shift from NHI to MA. Our study is the first in South Korea to compare the health care utilization and out-of-pocket spending of individuals who have NHI coverage and those who have shifted from NHI to MA and have similar SES.

Therefore, in our study, we identified a case group that has experienced a shift in coverage from NHI to MA because we mainly hypothesized that becoming MA beneficiaries could lead higher use of health care utilization and spending, compared with a matched control group that has had consecutive NHI coverage and exhibits SES characteristics similar to the case group. Subsequently, we were able to estimate differential changes in the groups’ health care utilization and out-of-pocket medical spending in the pre-and post-shift periods using a difference-in-differences (DiD) analysis method. We also hypothesized that the shift in coverage would increase both the number of outpatient visits and length of stay (LOS) and decrease out-of-pocket medical spending.

## Methods

### Data source

We analyzed data from the Korean Welfare Panel Study (KoWePS), 2010–2016 conducted by the Korean Institute for Health and Social Affairs. The KoWePS data are nationally representative as stratified multistage probability sampling to select households from rural and urban areas was employed. All family members of both parents and children in the selected households were interviewed. Face-to-face interviews were conducted annually from January to February, using a computer-assisted personal interviewing technique. The KoWePS database includes detailed information about the respondents and their household members, including general characteristics, social security status, health care utilization patterns, economic and demographic backgrounds, subjective health status, and behavioral health status.

### Difference-in-differences study design

When examining the impact of an intervention or change in policy, the challenge is in determining whether the observed changes are attributable to the intervention. A valid method of assessing this is to compare outcomes for the group that is subject to the intervention (the case group) with a group that is not (the control group). As a randomized study design is rarely feasible in the field of health policy, a quasi-experimental study design to measure the effect of health care interventions is frequently applied.

We employed an observational study along with a DiD analysis, which is a widely used quasi-experimental study design, to compare health care utilization and out-of-pocket medical spending between the case group and its matched control group. To do so, we began by matching characteristics between the groups and controlled for background trends by performing a propensity score (PS) match.

The DiD approach necessitates some assumptions to evaluate the intervention effect accurately. First, as the DiD estimator measures the treatment effect by examining the difference in the average outcome between the control and case groups, before and after treatment [14], at least two periods of data must be available for each group. Second, the DiD approach is valid only if there are no underlying time-dependent trends in the outcomes that are unrelated to the change of coverage [14]. If the outcomes were already improving before the shift, then a pre−/post-study would erroneously conclude that the policy was associated with better outcomes [14]. The DiD study addresses this problem by using a comparison group that is experiencing the same trends but which is not exposed to the policy change; this is also known as the parallel trend assumption [15, 16]. Third, the control group should be the same as the intervention group in everything other than the change in policy [14, 17, 18]. In practice, however, observed and unobserved differences exist between the two groups. To minimize the differences, we applied the PS matching technique. We assumed that in the absence of the policy intervention, the unobserved differences between the two groups would converge over time [17, 18].

### Propensity score matching and covariate selection

PS matching aims to find one or more individuals with a similar PS from the treatment and control groups. Various methods are employed to match individuals, but we used a 1:3 nearest-neighbor matching method which matches case and control individuals who have a similar propensity score value [19]. We added constraint that the difference between the PS (caliper width) should be 0.1 at most, to avoid pairing dissimilar individuals. We also considered methodological theories for selecting the covariates in the PS model. First, using prior knowledge to identify the covariates that affect the outcome and including them in the PS model is better for estimating the intervention effect [20]. Second, selecting covariates that are strongly associated with exposure but unrelated to the outcome should be avoided, because this may increase the bias. Selecting variables for the PSs, based on their association with the outcome may help to reduce such a bias [21]. Therefore, to estimate the medical coverage shift effect, we matched the control group to the case group by including the following parameters or questions in the PS model: gender; residential area (in the capital or elsewhere); marital status (yes or no); economic activity (yes or no); age (< 20, < 40, < 65, or ≥ 65 years); equivalized household disposable income (quintile groups; Q1–Q5); subjective health status (good, moderate, or bad); the number of private insurance schemes; expenditure on private insurance; and survey year (2010–2016).

### Intervention

The shift from NHI to MA Type I or II was represented by the intervention variable. Based on the intervention time, we classified the pre- and post-intervention periods. Then, we classified the available data for the case group into models according to the following periods: from 1 year before and after (Model 1); 2 years before and after (Model 2); and 3 years before and after (Model 3) (Figure S1). A potential critical issue is that the intervention time may differ among the individuals in the case group. However, since we matched the survey years along with the SES variables between the case and control groups, we could match the individuals precisely in the case and control groups for each year.

### Study population

**Fig. 1:**
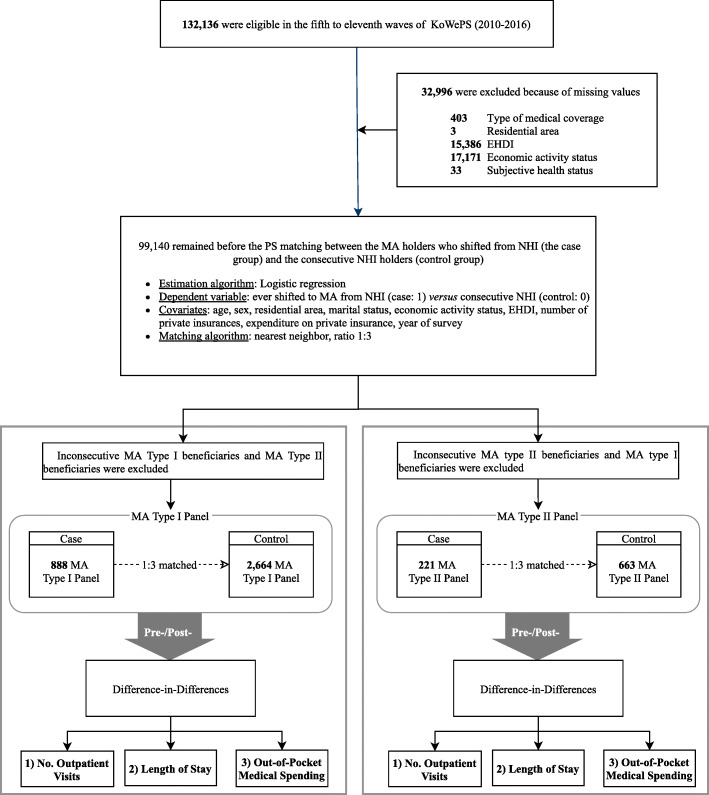
Flowchart of the study design. Abbreviation: Korean Welfare Panel Study: KoWePS; Equivalized Household Disposable Income: EHDI; Propensity Score: PS; Medical Aid: MA

We initially included 132,136 individuals from the KoWePS dataset of 2010–2016. After excluding those with missing values, 99,140 remained. We separated these into the MA Type I panel, which included 888 MA Type I beneficiaries and 2664 matched controls; and the MA Type II panel, which contained 221 MA Type II beneficiaries and 663 matched controls (Fig. 1).

### Outcomes

We examined changes in the individuals’ average health care utilization and out-of-pocket medical spending in the previous year. The first outcome was the differential changes between the groups in the average number of outpatient visits annually per person in the pre- and post-intervention periods. The second outcome was the differential changes in the LOS, and the third was the differential change in the average out-of-pocket medical spending.

### Statistical analysis

We used the generalized estimating equation (GEE) and DiD approach to estimate the changes in health care utilization and out-of-pocket spending from the pre- to the post-intervention periods that differed from concurrent changes in the case group and its matched control group. The GEE model accounts for time variations and correlations among repeated measurements that are present in longitudinal study designs and is appropriate for marginal estimates with non-linear link functions [22]. We applied the log-link with zero-inflated negative binomial distribution to the outcome variables because of the high incidence of zero counts in outpatient visits, LOS, and out-of-pocket medical spending [23]. Then, specifically, for each dependent variable, we fit the following model:

$$ \log \left(\mathrm{E}\left({\mathrm{Y}}_{it}\right)\right)={\upbeta}_0+{\upbeta}_1\ \mathrm{case}\_{\mathrm{indicator}}_i+{\upbeta}_2\ \mathrm{post}\_{\mathrm{indicator}}_t+{\upbeta}_3\ \left(\mathrm{case}\_{\mathrm{indicator}}_i\times \mathrm{post}\_{\mathrm{indicator}}_t\right)+{\upbeta}_4\ {\mathrm{covariates}}_{it}, $$

where Log(E) denotes the exponentiated expected value; Y_*it*_ is the initial outcome with a specified distribution option (health care utilization and out-of-pocket spending) for individual *i* at time *t* from intervention; case_indicator is a vector of the groups (case or control group); post_indicator is a vector of the pre−/post-indicators (whether individual *i* entered the post-intervention or not); covariates denote a vector of the other individual’s characteristics (the most-visited type of medical institution and last period of affliction by chronic disease). All the statistical tests were two-tailed, and a *p-value <* 0.05 was considered significant; analyses were performed using the Statistical Analysis System version 9.4 (Cary, North Carolina, USA).

## Results

### Characteristics of the study population

Eight hundred eighty-eight beneficiaries underwent a shift in medical coverage from NHI to MA Type I, and 221 beneficiaries experienced a shift in medical coverage from NHI to MA Type II. By considering their SES characteristics and the survey year, the matched control groups were selected through a 1:3 PS matching process (details of the PS matching process are included in the Methods section and shown in Fig. 1). The bivariate result showed that there were no significant differences in any year, among the individuals in the case and control groups (Table 1).

**Table 1:** Study Population Characteristics Among National Health Insurance Holders and Those Who Shifted From National Health Insurance to Medical Aid Who Were Matched Using the Propensity Score Before and After the Shift For All Years

	Medical Aid Type I Panel				Medical Aid Type II Panel			
	NHI→Medical Aid Type I	Consecutive NHI		***P***-value after Matching^**a**^	NHI→Medical Aid Type II	Consecutive NHI		***P***-value after Matching^**a**^
		Before Matching	After Matching			Before Matching	After Matching	
**No. of subjects**	888	17,629	2664		221	17,629	663	
**Subjects’ characteristics**								
**Sex, women, %**	61.0	53.9	60.8	.94	62.5	53.9	60.3	.57
**Residential area, in capital, %**	32.0	37.6	31.5	.79	25.9	37.6	24.5	.68
**Married, %**	46.2	60.1	47.1	.64	32.4	60.1	36.1	.32
**Economic activity, %**	25.0	48.9	24.6	.81	40.7	48.9	40.9	.97
**Age, %**								
< 20	2.4	4.4	2.1	.94	9.3	4.4	8.2	.53
< 40	7.4	37.1	7.5		13.0	37.1	17.1	
< 65	19.3	36.9	18.9		41.2	36.9	39.2	
65 ≤	70.9	31.7	71.5		36.6	31.7	35.5	
**Equivalized disposable household income**^**b**^**, %**								
Quintile 1 (0–20%)	73.2	18.4	73.6	.78	65.3	18.4	67.9	.50
Quintile 2 (21–40%)	16.7	15.6	16.0		30.1	15.6	25.3	
Quintile 3 (41–60%)	6.0	15.5	5.6		3.2	15.5	4.9	
Quintile 4 (61–80%)	2.3	20.3	3.1		0.9	20.3	1.5	
Quintile 5 (81–100%)	1.8	30.2	1.7		0.5	30.2	0.3	
**Subjective health status, %**								
Good	30.9	60.0	30.5	.96	54.6	60.0	56.0	.94
Moderate	23.9	17.9	23.8		19.9	17.9	19.4	
Bad	45.2	22.1	45.7		25.5	22.1	24.5	
**No. of private insurances, mean (*SD*)**	0.1 (0.5)	0.8 (1.8)	0.1 (0.5)	.23	0.3 (0.6)	0.8 (1.8)	0.3 (0.7)	.29
**Expenditure on private insurance, $/year, mean (*SD*)**^**c**^	253.3 (1065.0)	1520.1 (2234.1)	208.2 (844.0)	.25	429.5 (867.7)	1520.1 (2234.1)	490.3 (1042.8)	.40
**Year, %**								
2010	12.8	12.2	12.6	1.00	13.9	12.2	15.0	.96
2011	12.7	12.5	12.8		16.2	12.5	18.5	
2012	17.7	15.8	17.5		21.3	15.8	19.9	
2013	15.2	15.6	15.6		19.0	15.6	17.3	
2014	15.8	15.1	15.7		15.7	15.1	15.7	
2015	13.7	14.8	13.6		9.7	14.8	8.6	
2016	12.1	14.1	12.2		4.2	14.1	4.9	

*Abbreviation*: *NHI* National Health Insurance, *SD* Standard Deviation

^*^Table 1 represents the characteristics of the subjects in the Medical Aid Type I panel and Medical Aid Type II panel

^a^*P*-values reflect t-tests for continuous variables (number of private insurances and expenditure on private insurance) and x2 tests for dichotomous/categorical variables (age, sex, equivalent disposable household income, marital status, economic activity status, and subjective health status

^b^All of the subjects’ equivalent household income levels were allocated into each quantile for each year based on the data from the Korean government’s statistics report

^c^Calculated in dollars ($) from won (₩) at the exchange rate announced every December in the Korean statistics

### Pre-intervention trends

**Fig. 2:**
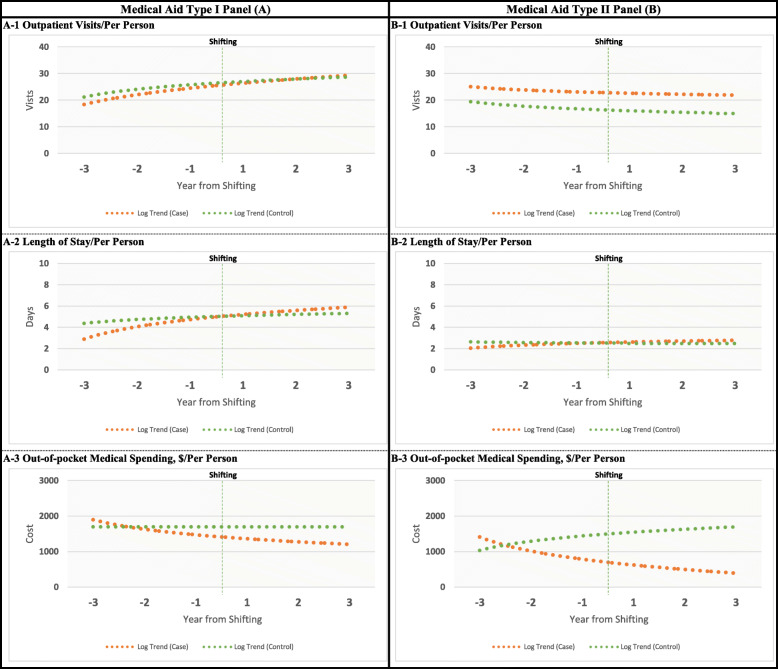
Trends in unadjusted health care utilization and out-of-pocket medical spending among NHI holders and those who shifted from NHI to MA for the Medical Aid Type I panel (A1, 2, and 3) and MA Type II panel (B1, 2, and 3)

The trends in unadjusted health care utilization and out-of-pocket medical spending of the MA beneficiaries and NHI holders throughout the study period are shown in Fig. 2. In the MA Type I and II panels, the trends in the average number of outpatient visits and LOS among the case and control groups before the intervention were parallel, but out-of-pocket medical spending was not. Given the significant difference in trends between the two groups and the possibility of a bias in the analysis [14], it was deemed appropriate to address the estimates of the number of outpatient visits and LOS in both panels, but not the estimates of out-of-pocket medical spending.

### Health care utilization [outpatient visit, length of stay]

In the MA Type I panel, in Model 1, the average number of outpatient visits increased from 25.7 times per year to 34.6 times per year in the case group, and it was almost the same in the control group. The adjusted differential change between the case and control groups for Model 1 is 31.8%, *p* < 0.021. The results remained statistically significant in Model 2 and Model 3 at 22.3%, *p* < 0.032 and 18.8%, *p* < 0.044, respectively (Table 2, Table S1). However, differential changes in LOS were not found in any of the periods. Regarding the MA Type II panel, there were no significant differential changes in any health care utilization (Table 2, Table S2).

**Table 2:** Differential Changes in Health Care Utilization and Out-of-Pocket Medical Spending by the Medical Aid Type I and II Panels

Health Care Utilization	Mean (***SE***)						Adjusted Difference-in-Differences Estimates, % (95% CI)^**b**^	***P***-value
	Case Group^**a**^		Control Group^**a**^					
	Before Shifting Period	After Shifting Period	Unadjusted Difference	Before Shifting Period	After Shifting Period	Unadjusted Difference		
**Medical Aid Type I Panel**								
**1 year before and after (Model 1)**								
**Outpatient Visit/per person**	25.7 (2.8)	34.6 (2.9)	8.9	28.4 (1.6)	28.0 (1.7)	−0.4	31.8 (4.2 to 66.8)	0.021
**Length of Stay/per person**	8.5 (1.5)	6.9 (1.6)	−1.6	3.3 (0.9)	5.5 (0.9)	2.2	−40.7 (−72.6 to 28.1)	0.184
**Out-of-pocket medical spending, $/per person**^**c**^	1402.9 (157.9)	1272.9 (163.0)	− 130.0	1527.0 (91.2)	1803.3 (94.1)	276.3	−20.2 (− 38.7 to 3.7)	0.092
**2 years before and after (Model 2)**								
**Outpatient Visit/per person**	24.2 (2.1)	33.0 (2.2)	8.8	26.2 (1.2)	28.5 (1.3)	2.3	22.3 (1.7 to 47.0)	0.032
**Length of Stay/per person**	6.2 (1.2)	5.8 (1.2)	−0.4	3.9 (0.7)	5.6 (0.7)	1.7	−20.4 (−57.6 to 49.2)	0.476
**Out-of-pocket medical spending, $/per person**^**c**^	1423.7 (128.5)	1219.7 (135.9)	− 204.0	1574.4 (74.2)	1778.4 (78.5)	204.0	−15.7 (−32.3 to 4.6)	0.120
**3 years before and after (Model 3)**								
**Outpatient Visit/per person**	22.7 (1.9)	32.1 (2.0)	9.4	25.9 (1.1)	29.5 (1.1)	3.6	18.8 (0.5 to 40.5)	0.044
**Length of Stay/per person**	5.9 (1.1)	5.5 (1.1)	−0.4	4.4 (0.6)	5.6 (0.7)	1.2	−7.2 (−47.3 to 63.4)	0.795
**Out-of-pocket medical spending, $/per person**^**c**^	1500.5 (116.2)	1151.8 (122.6)	− 348.7	1621.6 (67.1)	1751.4 (70.8)	129.8	−23.2 (−36.9 to −6.5)	0.009
**Medical Aid Type II Panel**								
**1 year before and after (Model 1)**								
**Outpatient Visit/per person**	22.6 (4.1)	32.0 (4.2)	9.4	16.3 (2.4)	18.5 (2.4)	2.2	38.9 (−11.3 to 84.0)	0.151
**Length of Stay/per person**	4.4 (2.5)	5.7 (2.6)	1.3	2.9 (1.5)	3.3 (1.5)	0.4	61.7 (−80.5 to 1330.4)	0.656
**Out-of-pocket medical spending, $/per person**^**c**^	880.0 (233.9)	816.4 (237.9)	−63.6	1246.8 (135.0)	1605.2 (137.3)	358.4	−31.8 (−57.4 to 9.3)	0.112
**2 years before and after (Model 2)**								
**Outpatient Visit/per person**	24.3 (3.3)	27.7 (3.6)	3.4	16.8 (1.9)	17.3 (2.1)	0.5	25.0 (−15.0 to 84.0)	0.257
**Length of Stay/per person**	3.0 (1.9)	4.7 (2.1)	1.7	2.7 (1.1)	2.8 (1.2)	0.1	48.2 (−80.5 to 1124.0)	0.704
**Out-of-pocket medical spending, $/per person**^**c**^	711.5 (190.1)	728.1 (211.4)	16.6	1278.1 (109.8)	1676.4 (122.0)	398.3	−22.7 (−48.8 to 16.5)	0.219
**3 years before and after (Model 3)**								
**Outpatient Visit/per person**	24.3 (2.8)	26.1 (3.3)	1.8	16.6 (1.6)	16.2 (1.9)	−0.4	38.6 (−2.7 to 97.6)	0.071
**Length of Stay/per person**	2.5 (1.5)	4.4 (1.8)	1.9	2.6 (0.9)	2.8 (1.0)	0.2	221.1 (−66.8 to 1471.3)	0.412
**Out-of-pocket medical spending, $/per person**^**c**^	668.6 (166.0)	656.1 (196.2)	−12.5	1321.1 (95.8)	1641.6 (113.3)	320.5	−23.9 (−47.7 to 10.8)	0.154

*Abbreviation*: *SE* Standard Error, *CI* Confidence Interval

^a^Propensity score matched for sex, age, region, equivalent household income, marital status, economic activity status, number of private insurances, and expenditure on private insurance

^b^All estimates were adjusted for the individuals’ fixed characteristics (most visited type of medical institution and last period of chronic disease). A positive sign corresponds to a larger increase in utilization over the study period among Medical Aid-shifted recipients, compared with National Health Insurance holders

^c^Calculated in Dollar($) from Won(₩) at the exchange rate announced by the Korean Statistics yearly in every december

### Out-of-pocket medical spending

In both the MA Type I and II panels, there were no significant changes in out-of-pocket medical spending. Even though the differential change in out-of-pocket medical spending in Model 3 was statistically significant (adjusted differential change between the case and control group: − 23.2%, *p* < 0.009), it is not appropriate to interpret it as meaningful because a parallel trend was not found in the pre-intervention period.

## Discussion

Using the DiD approach, we estimated the effect a shift in medical coverage has on health care utilization and medical spending. We compared a case group with a control group with similar SES and subjective health status parameters. We found that a shift in medical coverage from NHI to MA Type I increased outpatient visits but did not affect LOS. Furthermore, we did not find differential changes in out-of-pocket spending between the case and control groups in the MA Type I panel. Thus, we can state there was an increase in outpatient visits but no changes in out-of-pocket spending.

Our results deviate from the findings of previous studies as we did not compare MA beneficiaries with the general population but instead with a population subset with similar characteristics. We estimated the differential changes in the outcomes between the pre-shift period and post-shift period; accordingly, different results were found.

Unlike the findings from previous studies that MA beneficiaries remain in hospital longer than the general population [24, 25], we found that the shift to MA did not increase LOS significantly when compared to groups with similar characteristics. Furthermore, by examining MA Type I and II, we identified that the shift to MA Type I could induce frequent outpatient visits, but the shift to Type II did not. The reasons for these results are as follows: (1) Type II beneficiaries are subject to higher amounts of out-of-pocket spending on health care because Type I are subject to little or no copayment, and Type II are subject to 10–15% copayment of the medical costs. (2) MA Type II beneficiaries are more likely to be healthier than Type I beneficiaries since most of them are younger and able to work.

Approximately 10% of the total population of South Korea is 120% below the poverty line; of these, only about 3% are MA beneficiaries, and the rest are unacknowledged by the health care system receiving little medical cost assistance from the government [26–28]. This is a challenging issue as this part of the population remains exposed to health risks due to poor access to health care services [26], and is a moral hazard for MA beneficiaries (especially Type I) [13, 24]. This indicates that limited government finances are not being spent efficiently.

Previously, the government has implemented policies and programs to address these issues, including mandating the outpatient copayment system for Type I beneficiaries, introducing a monthly health management fee to regulate possible abuse of medical utilization, and a case management program [9]. However, they have not been very successful [9–11, 29–31]. The reasons are as follows: (1) weak government control over medical access by beneficiaries [9, 11, 29], (2) little or no copayment fee [8–13, 25, 30], and (3) selection criteria for MA beneficiaries that focuses more on family characteristics than individual characteristics [29].

A major reason for the continuous, excessive use of medical services is that the minimum level of cost-sharing is too low [8–13, 25, 30]. Increasing the minimum level or converting the copayment system to coinsurance or deductible payments in the MA Type I could be considered. Although the suggestion may be controversial because it would impose an additional burden on some beneficiaries, we support it for the following reasons. First, adequate copayment or coinsurance payment is associated with a decrease in unnecessary medical utilization; a similar step was implemented in the USA’s healthcare system. In the USA, public insurance for the lowest income population with little copayment or coinsurance has been a concern since it burdens the fiscal with excessive use of medical services along with moral hazards. An empirical example is Massachusetts’ Commonwealth Care program, which imposed higher copayments to low-income enrollees to reduce the fiscal pressures associated with insurance expansion by the scope for moral hazard [32]. However, there remained speculation that low-income populations may be more likely to have adverse health consequences from the result of higher cost-sharing as they could not afford the increased burden, which is also called “offset effects” [32, 33]. However, the evidence proved that no notable offset effect was found with the increase of copayment in the low-income population [33]. Accordingly, imposing copayment for the use of the emergency department [34], or pharmaceutical services in a Medicaid program [35, 36] reportedly improved overall health status. Second, the degree of cost-sharing has changed little since it was introduced in 2007. Third, excessive government expenditure on MA reduces the availability of funds for allocation to other governmental medical support schemes. A statistical report from the Korean government has indicated that MA payments account for 12.0% of the total NHI expenditure, and the total medical expenses related to MA increased dramatically by 39.2% (from 5.1 trillion Won in 2011 to 7.1 trillion Won in 2017). This was despite a decrease in the number of MA beneficiaries from 1.61 million people to 1.49 million people (− 7.65%) during the same period [37]. Furthermore, compared to NHI holders, the MA beneficiaries’ medical expenses per capita is 3.6 times higher, and the LOS per capita is four times higher [37]. Considering that MA beneficiaries are a vulnerable population, 22.9% of the entire population of MA beneficiaries used health care for more than 365 days (the total number of days for outpatient visits, LOS, and medication dosage) [37]. This suggests that adequate control of medical utilization by MA beneficiaries is still lacking. Additionally, our study implies that being an MA beneficiary significantly increases the number of outpatient visits without any significant changes to out-of-pocket spending on medical use. From previous studies, we can infer that the reason newcomers to MA frequently use outpatient services is that the out-of-pocket spending level is extremely low [8–13, 25, 30]. Therefore, a modification of the MA cost-sharing level should be discussed thoroughly with evidence-based future researches.

As our study has several limitations, the results should be interpreted and generalized with caution. First, owing to data limitation, we could not incorporate several other factors that may affect health care utilization. In the DiD approach, however, if the parallel trend assumption is met between the case and control groups, it can remove the effect of any confounding factors [14–16]. Therefore, the estimates of the number of outpatient visits and LOS in the MA Type I and Type II panels could be free from confounding issues to some extent. Second, we were not able to consider the number of emergency visits separately since there was no such information in the KoWePS data. Third, the KoWePS data obtained information about health care utilization through self-reporting, and the surveyors collected data retrospectively based on receipts. Therefore, these two factors may have distorted the results of medical utilization.

## Conclusions

Regarding the ongoing problem of excess medical utilization by MA Type I beneficiaries in Korea, restraint should be in place. Therefore, our research provides evidence that the shift in medical coverage from NHI to MA Type I increased the number of outpatient visits without increasing the out-of-pocket spending. However, neither of increased outpatient visit and out-of-pocket spending was found for shifting from NHI to MA Type II. Further research requires in-depth discussions on the appropriateness of the current cost-sharing level on MA beneficiaries.

## Supplementary information

**Additional file 1: Figure S1**. Study design used for the analysis.

**Additional file 2: Table S1**. Estimated Average Health Care Utilization and Out-of-pocket Medical Spending Difference in Medical Aid Type I panel by difference-in-differences analysis. Abbreviation: Medical Aid, MA, National Health Insurance, NHI, Confidence Interval, CI. * Log Link with Zero-Inflated Negative Binomial Distribution was applied in the regression analysis because of exceess zeros in the outcomes. ^a^ Models additionally adjusted for individuals’ health related characteristics (mostly visited type of medical institution, lasted period of chronic disease). ^b^ Propensity score matched for sex, age, region, equivalized household disposable income, marital status, economic activity status, number of private insurance, expenditure on private insurance.

**Additional file 3: Table S2**. Estimated Average Health Care Utilization and Out-of-pocket Medical Spending Difference in Medical Aid Type II panel by difference-in-differences analysis. * Log Link with Zero-Inflated Negative Binomial Distribution was applied in the regression analysis because of exceess zeros in the outcomes. ^a^ Models additionally adjusted for individuals’ health related characteristics (mostly visited type of medical institution, lasted period of chronic disease). ^b^ Propensity score matched for sex, age, region, equivalized household disposable income, marital status, economic activity status, number of private insurance, expenditure on private insurance.

## Data Availability

The datasets used and/or analyzed during the current study are available from the corresponding author on reasonable request.

## Abbreviations

DiD: Difference in differences
EHDI: Equivalized household disposable income
GEE: Generalized estimating equation
KoWePS: Korean Welfare Panel Study
LOS: Length of stay
MA: Medical Aid
NHI: National Health Insurance
PS: Propensity score
SES: Socioeconomic status
