# Supplementary figures and images for: The effect of shifting medical coverage from National Health Insurance to Medical Aid type I and type II on health care utilization and out-of-pocket spending in South Korea
Source: BMC Health Serv Res. 2020 Oct 27;20:979. doi: 10.1186/s12913-020-05778-2 (PMC7590487; doi:10.1186/s12913-020-05778-2)

## Slide 1
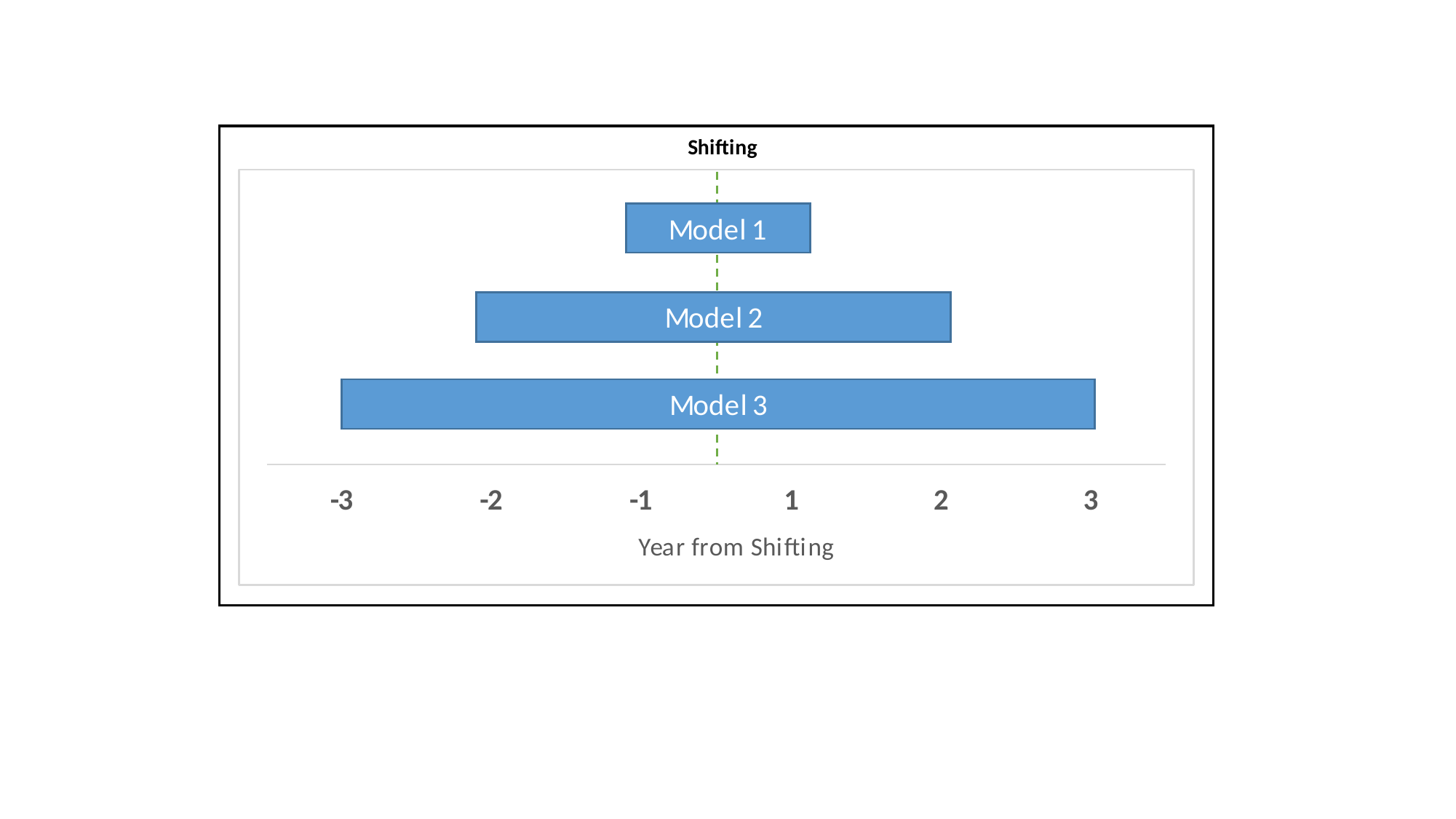

Supplement: Supplementary file 1 — Additional file 1: Figure S1. Study design used for the analysis. [file 12913_2020_5778_MOESM1_ESM.pptx]
